# Laparoscopic monolateral suspension for vaginal vault prolapse: a report of an exit surgical strategy during sacralcolpopexy

**DOI:** 10.1186/s12893-020-00861-1

**Published:** 2020-09-11

**Authors:** Federico Romano, Andrea Sartore, Denise Mordeglia, Giovanni Di Lorenzo, Guglielmo Stabile, Giuseppe Ricci

**Affiliations:** 1 Institute for Maternal and Child Health, I.R.C.C.S. “Burlo Garofolo”, Trieste, Italy; 2 Department of Medical, Surgical, and Health Sciences, University of Trieste, Trieste, Italy

**Keywords:** Laparoscopic lateral colposuspension, Osteophytosis, Promontory dissection, Sacralcolpopexy, Vaginal vault prolapse

## Abstract

**Background:**

Vaginal vault prolapse is the most frequent long-term complication in patients undergoing hysterectomy and sacralcolpopexy is considered the gold standard. We report our surgical strategy maintaining single-arm mesh when the sacral promontory is not accessible to fix the mesh for an unknown sacral osteophytosis during a laparoscopic sacralcolpopexy. This is significant because, to our knowledge, the bone variant as a procedure limiting factor has never been described before. This opens new horizons for the sacralcolpopexy surgery, because it becomes necessary to know of a valid surgical alternative with mesh maintenance if this complication occurs again or to perform an assessment of the accessibility of the sacral promontory immediately after its dissection.

**Case presentation:**

We present a case of a 75-year-old woman with recurrence of vaginal vault prolapse. A laparoscopic sacralcolpopexy was recommended. During surgery, we found that the procedure was not feasible due to the presence of an unknown osteophytosis of the sacrum which prevented the fixing of the mesh to the sacral promontory. We decided to proceed with a single-arm lateral suspension by using a modified approach of the original technique, maintaining the mesh originally shaped for the sacral colpopexy. At follow-up, the vaginal vault is well suspended.

**Conclusion:**

This exit strategy may represent a valid surgical alternative when laparoscopic sacral colpopexy is not possible for anatomical variants, allowing to keep the laparoscopic approach using mesh. To our knowledge, cases in which the anatomical bone variant prevented access to the sacral promontory have never been described in the literature, as bone evaluation has never been considered a limiting element of this procedure.

## Background

Vaginal vault prolapse is the major long-term complication in patients undergoing hysterectomy and occurs, approximately, in 0.2–43% of the cases. The cause is the detachment of the pubocervical and rectovaginal fascia from each other, from their apical support, the uterosacral and cardinal ligaments or DeLancey’s level first support. The clinical management of this pathology remains a critical field, as demonstrated by the broad spectrum of surgical techniques developed so far [1].

The literature considers the sacralcolpopexy as the gold standard for vaginal vault prolapse, with a long-term success rates of > 90% and the most appropriate reconstruction of the physiological axis of the vagina [2]. Abdominal sacralcolpopexy has long been considered a safe and successful surgical procedure for the prolapse of the vaginal vault and has become the gold standard of care. The surgical approach may be abdominal by laparotomy, laparoscopy or robot-assisted [3]. Laparoscopic surgery combines the advantages of a repair, which is identical to the open transabdominal technique, with the advantages of vaginal surgery. Theoretically, all pelvic repair methods requiring laparotomy can also be performed with laparoscopy, which offers an excellent overall approach in the treatment of vaginal vault prolapse. It allows better visualization of the anterior and posterior pelvic anatomy obtained by pneumoperitoneal pressure which leads to a satisfactory repair [1]. Laparoscopic procedures have the disadvantage of having a longer operating time, which improves with the surgeon’s experience [4].

We report our experience which can represent a valid surgical alternative when the sacral promontory is inaccessible and the mesh is already fixed during laparoscopic sacralcolpopexy.

## Case presentation

We present the case of a 75-year-old woman who underwent a laparoscopic monolateral suspension for vaginal vault prolapse. The patient was a Caucasian woman in good general health with two previous spontaneous deliveries and a vaginal hysterectomy for uterine prolapse in 2001.

She was admitted to our hospital with a diagnosis of a recurrence of stage 4 of vaginal vault prolapse following the Pelvic Organ Prolapse Quantification (POP-Q) System, without stress urinary incontinence. The main symptoms she reported were a sense of weight and vaginal pressure. Considering the anamnesis, pelvic examination, clinical symptoms, and recurrence of pelvic organ prolapse, the surgical procedure chosen was a laparoscopic sacral colpopexy. The patient received adequate information on the surgical technique via laparoscopic access and, in case needed, via laparotomic or vaginal access.

A consent form was provided and signed. During surgery, the parietal peritoneum was opened at the level of the sacral promontory with subsequent incision of the presacral fascia and identification of the longitudinal ligament. After the visualization of the right ureter and homolateral hypogastric inferior right nerve [3], we performed a recto-vaginal dissection toward the perineal and anorectal junction together with dissection of the vesicovaginal space until the endopelvic fascia.

In our surgical practice, a 30 × 30 cm polypropylene mesh (Restorelle XL- Coloplast A/S, 3050 Denmark) with a single arm is used. Before its insertion in the abdominal cavity, the mesh is tailored only in the portion that will be anchored to the vaginal and rectal space, while the other end is left intact in its length and subsequently tailored after its attachment to the sacral promontory.

**Fig. 1:**
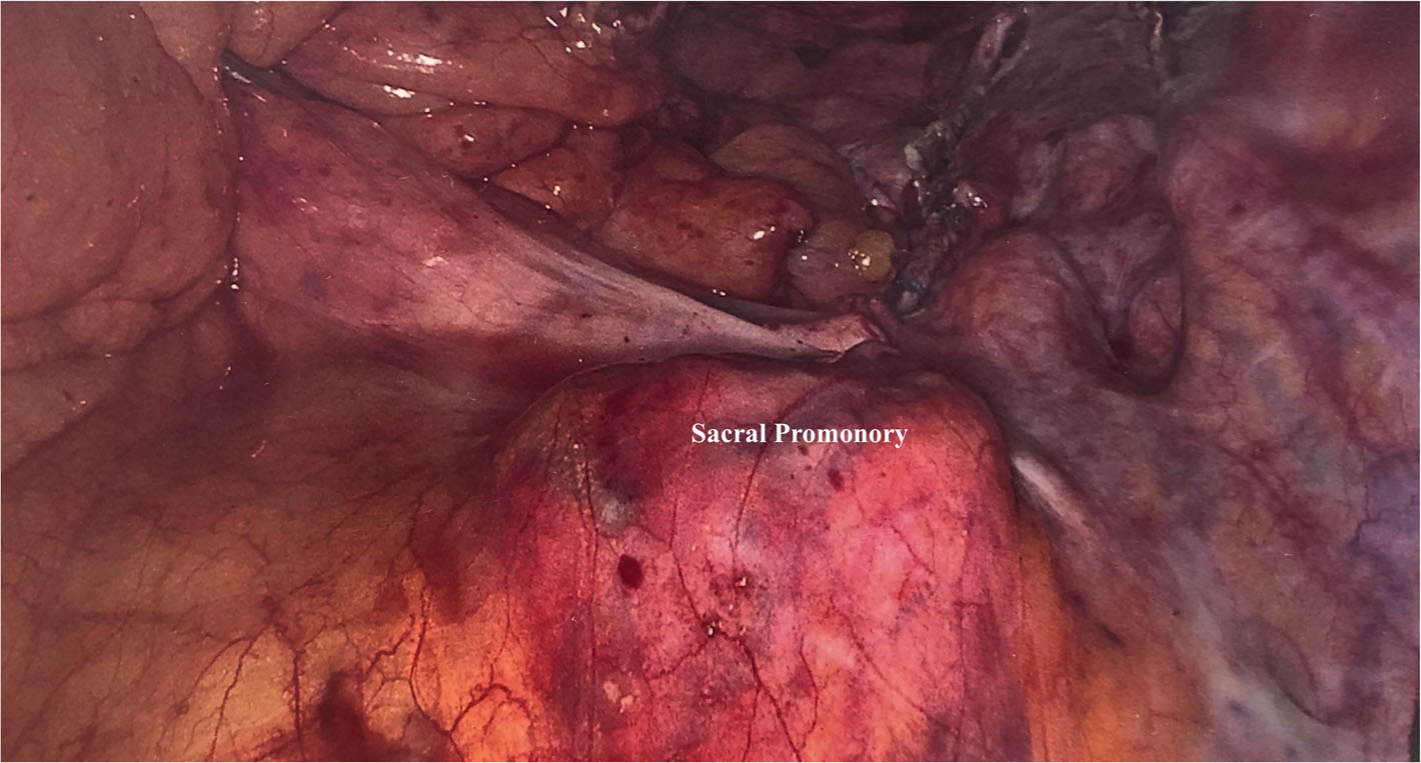
Laparoscopic image: sacral promontory

After the mesh was anchored on the vesical-vaginal space with 12 interrupted sutures (Ethibond 3–0 needle), multiple attempts to fix the mesh to the sacral promontory were tried firstly with an Ethibond 0 needle and then with non-absorbable helicoidal clips (5 mm CapSure–Permanent fixation System. Davol Inc. Subsidiary of C.R. Bard, Inc. 100 Crossings Boulevard Warwick, RI 02886 USA) but any attempt to penetrate the site was unsuccessful (Fig. 1). Considering that the mesh was already fixed to the anterior and posterior vaginal compartment, we intraoperatively decided to convert the procedure into a modified laparoscopic lateral suspension (LLS) with a monolateral right mesh, on the basis of the original technique described by Dubuisson et al., where a V-shaped mesh is used [5]. We carried out a retroperitoneal tunnel using laparoscopic forceps through the right lateral access under transperitoneal visualization, putting in tension and fixing the mesh to the Camper fascia with two interrupted sutures, using an Ethibond 2–0 needle.

**Fig. 2:**
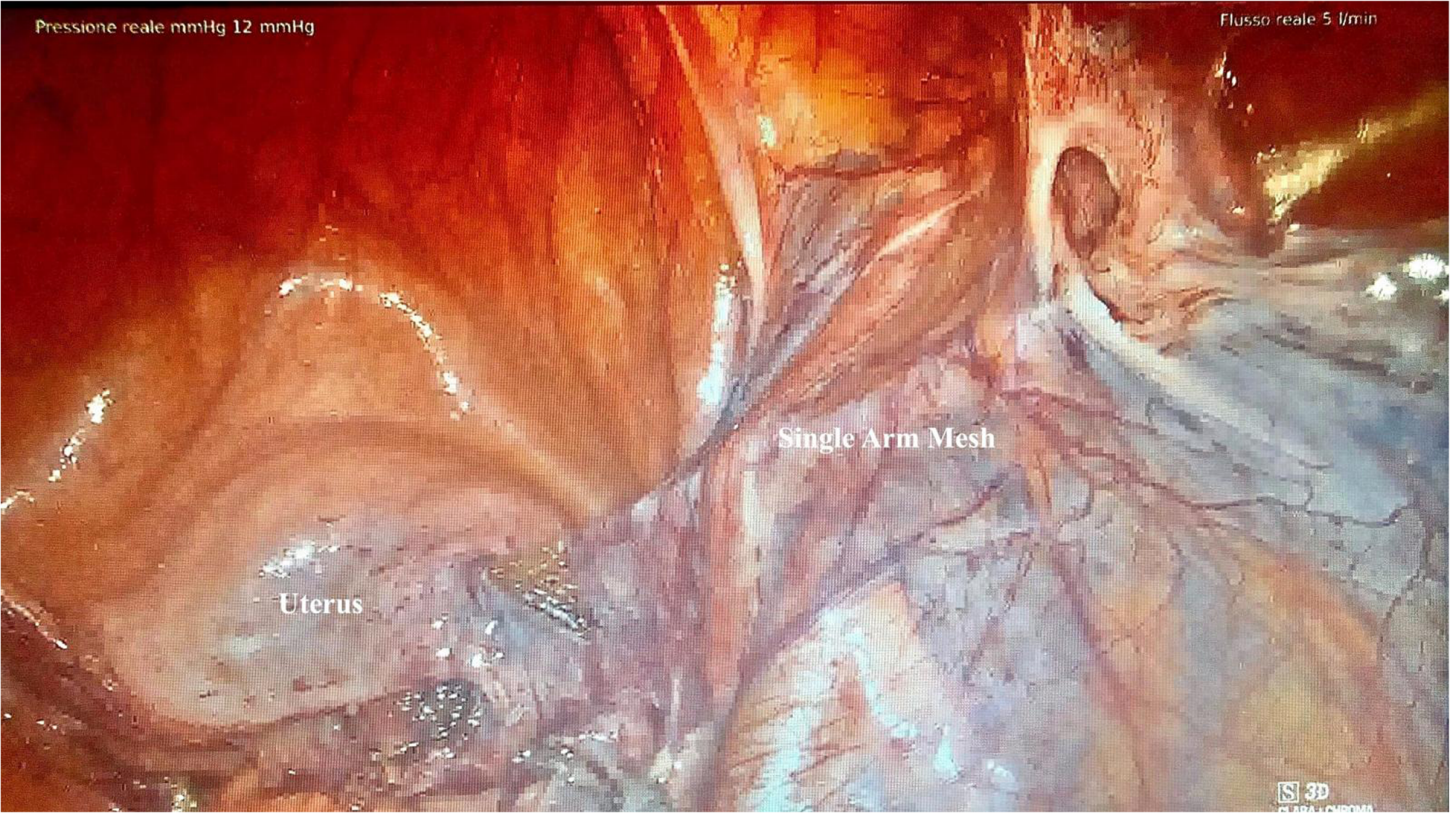
Laparoscopic image: lateral colposuspension of the mesh

At the end of the procedure, the vaginal vault was well suspended (Fig. 2). This strategy was possible because the sacral portion of the mesh was not shaped before its fixation to the sacral promontory and was long enough to allow its anchorage to the right lateral Camper fascia.

**Fig. 3:**
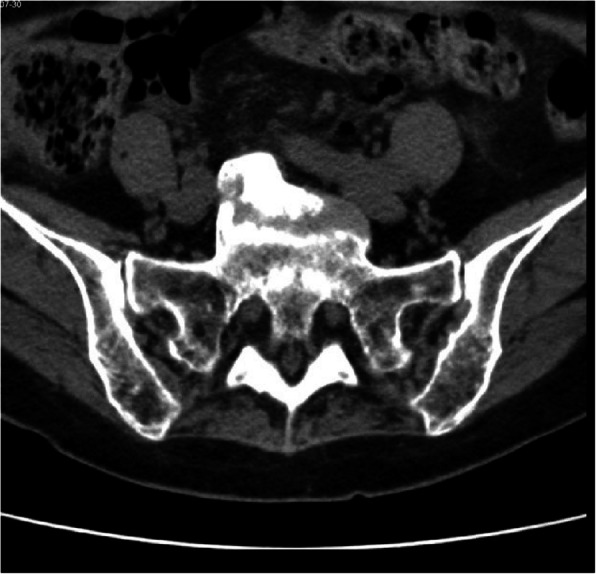
Transverse plane CT scan: sacral promontory

**Fig. 4:**
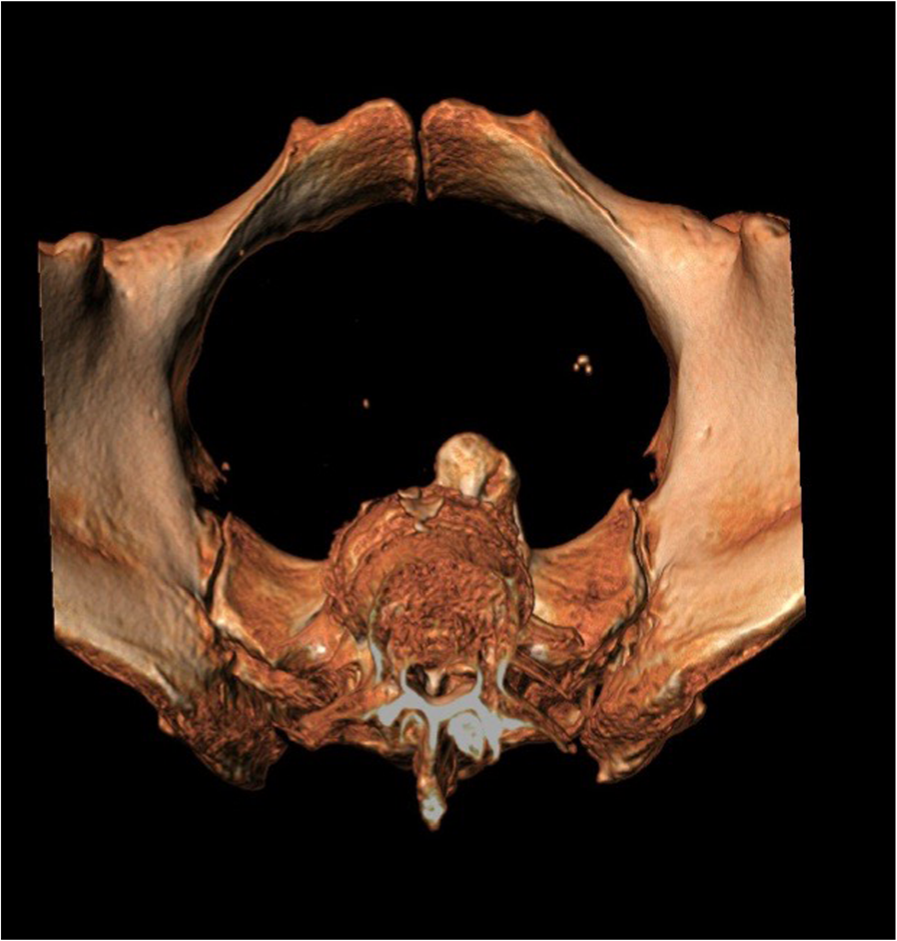
Transverse plane 3D CT scan: sacral promontory

During the post-operative recovery, we performed a 3D reconstruction CT scan to evaluate the sacrum which detected an exuberant osteophytic bridge at the level of the anterior right margin of L5 and S1 that protruded for 17 mm (Figs. 3 and 4).

The patient was discharged after 3 postoperative days with no post-surgical complication. At the follow-up visit, scheduled at 6 months after surgery, the vaginal vault was well suspended, with no signs of genital prolapse. The patient reported clinical well-being, without pelvic pressure or sensation of a vaginal bulge.

## Discussion and conclusions

Due to the surgical complexity and the high risk of injuring primary structures, the dissection of the promontory is considered the first surgical step [6]. The presacral area is defined as a retroperitoneal pyramid-shaped area that extends below the aortic bifurcation to the pelvic floor, bordered laterally by the internal iliac vessels. The peritoneal dissection generally begins from the sacral promontory, which represents an important bony landmark, and then extend downwards to expose the anterior longitudinal ligament [7]. The right ureter should be seen throughout the entire dissection process in order to avoid damages to the right iliac vessels and ureter. The vascular anatomy is extremely variable [8]. To date, the literature has strongly emphasized these variants which must be known to avoid bleeding complications, while the bone component and the study of the bone promontory were less explored.

An alternative procedure to the sacral colpopexy is the laparoscopic lateral suspension with a V-shaped mesh: this technique showed good results in terms of feasibility, postoperative results and patient’s satisfaction while avoiding the risks and difficulties related to the dissection of the promontory, requiring a shorter learning curve [9]. The choice of any of these procedures depends on patients’ clinical characteristics and surgical skills.

In our case, we were not aware of any previous traumatic event or clinical history that could raise the suspicion for anatomical variants of the sacral promontory and only postoperatively the patient referred a ski fall at a young age, that cannot be surely linked to the osteophyte. Moreover, the presence of the osteophytic bridge did not affect the normal dissection of the anatomical spaces and its presence was revealed only when we attempted to attach the mesh to the sacral promontory.

To our knowledge, this is the first case where an anatomical bone variant prevented to complete successfully the sacral colpopexy. Considering the rarity of this complication, it is not reasonable to recommend a radiological evaluation to all the patients who will undergo a laparoscopic sacral colpopexy. Therefore, our experience highlights two aspects: the first is the importance of carrying on a detailed clinical history in the preoperative time; the second, is to perform a tactile evaluation using laparoscopic instruments, which allow to obtain important information in the hands of experienced surgeons.

In case the anatomical variant is not detected before surgery, it is essential to think of an alternative strategy that allows to complete the procedure by keeping the laparoscopic approach and maintaining the mesh. Our modified laparoscopic monolateral suspension with a single-arm mesh, never described before, allowed us to overcome the surgical complication of an inaccessible promontory constituting a valid rescue surgical alternative.

Of note, a critical component of our surgical technique that allowed us to rapidly convert the approach was the possibility of using the mesh along its entire length. For this reason, a good surgical practice when approaching sacral colpopexy could be to shape the mesh longitudinally in width but not in length, modelling the proximal portion and cutting the distal end only after the completion of the fixation procedure.

We had a mid-term follow-up of 6 months that showed a good anatomical correction of the vaginal vault prolapse without relapse of the disease, despite the monolateral tension only on the right side. This aspect supports the feasibility of the described technique that could be of help for other surgeons facing similar anatomical variants.

The strengths of this case report are the feasible and safe reproducibility of this surgical procedure in case the sacralcolpopexy could not be completed with the possibility of maintaining the mesh and the laparoscopic surgical approach. The main limitation is that this procedure was performed on only one patient with short-term follow-up.

Our novel surgical technique consists in a laparoscopic monolateral suspension with a single arm mesh anchored only to one side of the Camper’ fascia. This approach, intraoperatively designed to overcome the unexpected finding of an anatomical bone variant, allowed us to complete successfully the surgery by keeping the laparoscopic approach and using the same mesh shaped for the originally procedure. Moreover, we highlight the importance of considering bone variants in the preoperative assessment of patients undergoing sacralcolpopexy, identifying different surgical strategies.

## Data Availability

Data sharing is not applicable to this article as no datasets were generated or analyzed during the current study.
